# Enhancing nucleic acid delivery by the integration of artificial intelligence into lipid nanoparticle formulation

**DOI:** 10.3389/fmedt.2025.1591119

**Published:** 2025-06-16

**Authors:** Kagya Amoako, Amir Mokhammad, Afrida Malik, Sumith Yesudasan, Anas Wheba, Oluwanifemi Olagunju, Sean X. Gu, Timur Yarovinsky, Edward Vincent S. Faustino, Juliane Nguyen, John Hwa

**Affiliations:** ^1^ Department of Chemistry and Chemical & Biomedical Engineering, University of New Haven, West Haven, CT, United States; ^2^ Department of Mechanical and Industrial Engineering, University of New Haven, West Haven, CT, United States; ^3^ Yale Cardiovascular Research Center, Section of Cardiovascular Medicine, Department of Internal Medicine, Yale University School of Medicine, New Haven, CT, United States; ^4^ Dept. of Laboratory Medicine, Yale University School of Medicine, New Haven, CT, United States; ^5^ Division of Pharmacoengineering and Molecular Pharmaceutics, Eshelman School of Pharmacy, University of North Carolina, Chapel Hill, NC, United States

**Keywords:** nucleic acid delivery, liposome nanoparticles, artificial intelligence, platelets, transfection

## Abstract

The advent of messenger RNA (mRNA) therapeutics has revolutionized medicine, with its potential underscored by rapid advancements during the COVID-19 pandemic. Despite its promise, nucleic acid delivery remains a formidable challenge due to enzymatic degradation, cellular uptake barriers, and endosomal trapping. Therapeutic lipid nanoparticles (LNPs), pioneered in the 1970s, have emerged as the gold standard for delivering mRNA and other nucleic acids, offering unparalleled advantages in stability, biocompatibility, and cellular targeting. This review explores the evolution and design of LNPs, focusing on their role in hematologic therapies and platelet transfection, where unique challenges arise due to platelets’ anucleate nature. The paper systematically evaluates the composition of LNPs, highlighting the role of ionizable, cationic, and neutral lipids in optimizing delivery efficiency, stability, and immune response modulation. Strategies to overcome platelet transfection barriers, including tailored lipid compositions and particle engineering, are discussed alongside advances in artificial intelligence (AI) for predictive nanoparticle design. Furthermore, it examines various nucleic acid cargoes, including mRNA, siRNA, and miRNA, and their therapeutic potential in addressing platelet-related disorders and advancing personalized medicine. Finally, the review delves into emerging technologies and the integration of AI to overcome existing barriers in nucleic acid delivery. By fostering interdisciplinary collaboration, this work aims to catalyze discoveries in LNP-based therapeutics and transformative advancements in hematologic treatments.

## Graphical Abstract

Overview of Lipid Nanoparticles (LNPs) for Nucleic Acid Delivery. Structure: A 3D-rendered schematic showing an LNP particle. Core: Encapsulated nucleic acid (e.g. mRNA) shown as a helical structure. Lipids: Layers illustrating cationic lipids (binding to nucleic acids) ionizable lipid (to aid endosomal escape), helper lipids, cholesterol, and PEG-lipids on the outer surface for circulation stability.

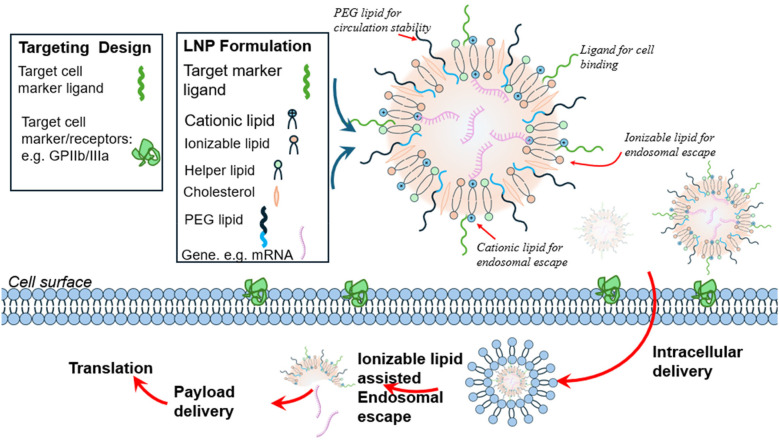

## 1 Introduction

**Figure 1:**
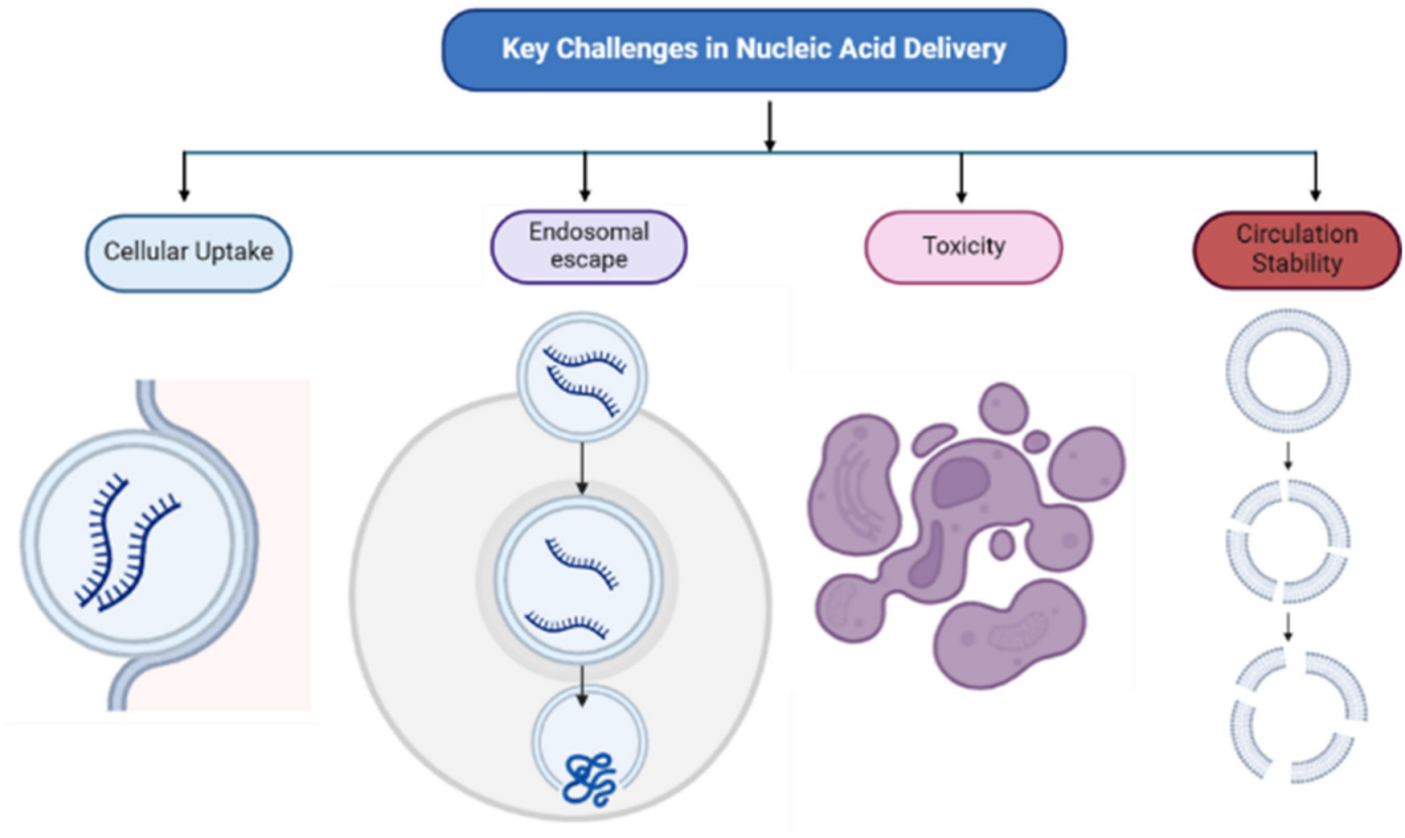
Key challenges in nucleic acid delivery: circulation stability, cellular uptake, endosomal escape, and toxicity. Nanoparticle degradation and rapid clearance by immune cells affect circulation stability while formulation composition influence cellular uptake, toxicity, and endosomal escape.

Nucleic acids are crucial biomolecules involved in genetic storage, transmission, and translation. Messenger RNA (mRNA) carries genetic information from DNA to the cytoplasm for protein synthesis. The recent success of mRNA-based COVID-19 vaccines stems from decades of foundational research. In the 1940s, the one gene-one enzyme hypothesis suggested genes encode enzymes, but the intermediary role of mRNA was unclear (1). Later research using radioactive labeling and centrifugation confirmed mRNA as distinct from DNA and ribosomal RNA, establishing its function in protein synthesis (2, 3). This discovery resulted from cumulative scientific efforts that advanced molecular biology. Unlike DNA, RNA therapies require delivery only to the cytoplasm, bypassing nuclear entry challenges. However, the naked RNA is rapidly degraded by nucleases and reactive oxygen species, reducing delivery efficiency (4–7). Additionally, immune recognition of free mRNA and its negative charge hinder cellular uptake (8–10). Even when internalized via endocytosis, mRNA often remains trapped in endosomes, requiring effective endosomal escape to reach the cytoplasm for protein translation. See Figure 1.

Research on improving nucleic acid transfection has focused on carrier materials to protect genetic material. The development of LNP-mRNA formulations in the 1970s was a breakthrough, as liposomes demonstrated the ability to encapsulate and shield nucleic acids from degradation, laying the foundation for LNP-based mRNA delivery (11–13). While DNA encapsulation research continues, mRNA delivery via liposomes has gained greater attention, leading to systematic evaluation of formulation strategies.

LNPs have proven superior to viral vectors and polymer-based carriers due to their ability to protect mRNA, enhance cellular uptake, and enable targeted delivery. Unlike viral vectors, LNPs are non-immunogenic and do not integrate into the host genome, reducing risks. Compared to polymer-based systems, LNPs offer better transfection efficiency and *in vivo* stability, making them the preferred choice for mRNA-based therapeutics, including COVID-19 vaccines (14, 15). LNPs' capacity to encapsulate RNA and facilitate uptake makes them a valuable tool in hematology, particularly for personalized medicine. Platelet-related disorders involve dysregulated protein function, affecting adhesion, aggregation, and clot formation. Mutations in glycoproteins like GPIb-IX-V or integrins cause bleeding disorders, while excessive activation of platelet proteins can lead to thrombosis (16, 17). Understanding these mechanisms is crucial for developing targeted mRNA therapies. mRNA delivery can transiently modify protein expression in target cells, making it ideal for vaccines, gene therapy, and cell-based treatments. By encoding therapeutic proteins, enzymes, or antibodies without requiring vector integration, mRNA-based approaches provide a flexible and efficient alternative to traditional protein therapies (18, 19).

Overall, the development of mRNA as a key player in cellular processes, particularly in protein synthesis, has revolutionized therapeutic strategies. The successful use of mRNA, especially with the aid of LNP formulations, has addressed significant challenges in gene delivery and expression, providing a versatile and effective platform for the treatment of various diseases, including hematological disorders. LNP-mRNA formulations offer critical advantages such as enhanced protection from degradation, efficient cellular uptake, and the ability to deliver therapeutic proteins directly to target cells. This has proven transformative in the development of vaccines and gene therapies and holds promise for personalized treatments in platelet-related diseases, where targeted modulation of protein expression can restore normal hemostasis and alleviate disease symptoms. As research continues to refine mRNA delivery systems, the potential for broader clinical applications, including the treatment of genetic and hematological disorders, continues to expand.

## 2 Overview of nanoparticle-based delivery systems

Nanoparticles serve as an effective encapsulating tool for mRNA, as they present barriers to mRNA degradation and enhance drug delivery efficacy (20). It can be composed of a variety of particle-based materials with different formulations with a nanometer size range of 10–300 nm diameter that improves the conventional way of delivering therapeutics (21). They can be designed to enhance cellular targeting by incorporating specific formulation features that focus on specific tissues and blood vessels. These targeted delivery approaches can address challenges related to drug distribution, initial metabolism, and uptake in a particular cell type. This advanced engineering method enables improved and precise delivery to areas that were previously unreachable by unbound drugs and molecules owing to their interactions. There are three major research areas for nanoparticles as encapsulating tools: Lipid-based systems, polymer-based systems (PNPs), metal- and metal oxide–based nanoparticles (22). Each of these nanoparticle-based delivery systems offer unique advantages around formulation composition material space, immunogenicity, bioavailability, and design for targeting. The disease target for which they are to be applied, and the mode of their administration are key guiding principles for the selection of the nanoparticle system.

### 2.1 Lipid-based nanoparticles

The current Covid-19 vaccine uses liposomal carriers which were the first nanocarriers with mRNA cargo receiving FDA approval (23). Lipid-based nanoparticles are generally spherical with a lipid layer and can be divided into liposomes and solid lipid nanoparticles (SLNs) or LNPs (24, 25). Liposomes feature at least one lipid bilayer, while LNPs have an outer lipid layer that may not form a continuous bilayer. These nanoparticles are ideal for skin applications due to the lipids in the stratum corneum, which are essential for skin integrity and hydration. Lipid-based NPs can deliver hydrophobic, hydrophilic, and lipophilic molecules based on their entrapment location within the NP, allowing for targeted outer shells and therapeutic inner cores for specific uses. However, liposomes encounter issues like increased uptake through macro and micropinocytosis, endosomal uptake, and faster clearance due to their resemblance to physiological liposomes and vesicles (26). Water-soluble drugs also rapidly leak from liposomal cores in blood. To mitigate these problems, liposomes are often modified with surface additions like polymers, peptides, or other materials to extend circulation time (27). LNPs generally include cationic lipids, ionizable lipids, or other lipids in their outer shell to encapsulate the chosen drug or molecule within their aqueous core, enhancing stability, compatibility, targeted delivery, and endosomal escape (28). Nanostructured lipid carriers (NLCs), a variant of SLNs, combine solid- and liquid-phase lipids, offering better loading capacity and stability than traditional SLNs (29).

### 2.2 Polymer-based nanoparticles

Polymer-based NPs in general comprise of various polymers to control their size and properties, reduce drug loss and premature degradation, and facilitate manufacturing and storage. Polymersomes, the polymeric counterparts of liposomes exhibit a robust and stable structure, attributed to the amphiphilic block copolymers that form their bilayer membrane. This structural integrity provides enhanced stability in various biological environments, ensuring that the encapsulated therapeutic agents remain protected until they reach their target. The polymeric outer shell protects the NPs from protein absorption and clearance by opsonins, extending circulation time or target site persistence (30). Biodegradable polymers used in biomedical applications degrade by hydrolysis and enzymatic cleavage, allowing controlled therapeutic release and eventual polymer disappearance. Polymeric NPs use polymer chains as the main agents, with common surface polymers including polyglycerols, polycyanoacrylate, and polyethylene glycol (PEG) (31). Block copolymers of poly(lactic-co-glycolic) acid (PLGA) and PEG can be used to formulate micelles with different core and surface properties. Hydrophobic polymers such as polylactic acid, polycaprolactone, and PLGA are frequently used in the formation of PNPs. PNPs can encapsulate therapeutics of various hydrophobicity within their core or on the surface. The formulation shape and therapeutic dispersion depend on the preparation method, with nanospheres having uniform dispersion and nanocapsules featuring a hollow core. Drug release kinetics depend on the drug, core matrix diffusion, pH, and other biological factors. *in vitro* studies often show controlled release, but this does not always predict *in vivo* release rates. PNPs, with their versatile surface and core modifications, are used to deliver drugs, nucleic acids, and other small molecules, including siRNA. Charged polymers like polyethylenimine are frequently used for loading nucleic acids due to their cationic nature, though this can cause cytotoxicity which can be addressed by reducing the cationic charge density (32).

## 3 Composition and functionality of lipid nanoparticles

### 3.1 Types of lipids used in LNPs

Essentially lipids are hydrophobic and are not soluble in water, however some types of lipids have a structure that allows them to become hydrophilic. For instance, every cell in mammalian organism has a cell membrane that consists of lipid bilayer (33). Some Lipid nanoparticles resemble this composition having lipids with hydrophobic tail and polar head. This amphiphilic structure is crucial for their function. Most common types of lipids used for nanoparticle formulation are ionizable lipids, cationic, phospholipids and others such as cholesterol (34).

Table 1 provides a concise summary of the major lipid categories used in LNP formulations, outlining their key properties, functional roles, representative examples, and supporting references. Ionizable lipids, which change charge based on pH, are critical for reducing toxicity in circulation and enabling endosomal escape—features exemplified by modern biodegradable lipids such as those used in mRNA vaccines (35, 36). Cationic lipids possess a permanent positive charge, allowing for strong binding to negatively charged mRNA; optimized forms like DOTAP, especially when combined with helper lipids like DOPE, enhance transfection efficiency while reducing cytotoxicity (37, 38). Phospholipids, naturally found in cell membranes, stabilize LNPs and support endosomal escape, with DSPC serving as a widely used helper lipid in FDA-approved and COVID-19 vaccine formulations (39–41). Cholesterol plays a structural role, reinforcing membrane stability and influencing organ-specific targeting, where both concentration and analog structure affect biodistribution (42, 43). PEG-lipids improve circulation time and prevent aggregation, with PEG chain length and composition modulating tissue-specific delivery and immune interactions, particularly in ocular applications (44, 45).

**Table 1:** Major lipid categories used in LNP formulations.

Lipid type	Key characteristics	Role in LNPs	Examples/notes	Refs
Ionizable Lipids	pH-sensitive, neutral at physiological pH, protonated in acidic endosome	Enhance biocompatibility, enable endosomal escape	DODAP, biodegradable ionizables (e.g., sphingomyelin); used in Moderna vaccines	(35, 36)
Cationic Lipids	Permanently positively charged, form complexes with mRNA	Facilitate mRNA binding, membrane fusion, and cell uptake	DOTMA, DOTAP, DOPE (helper lipid), PEG-Cer variants; marketed as Lipofectin, MegaFectin	(37, 38)
Phospholipids	Amphiphilic, found in natural membranes; often zwitterionic	Helper lipids for solubility, stability, and endosomal escape	DSPC used in patisiran, COVID-19 vaccines (Moderna, Pfizer)	(24 39–41)
Cholesterol	Rigid sterol, enhances membrane packing and LNP stability	Improves LNP integrity and targeting; can modulate biodistribution	20*α*-hydroxycholesterol improves liver cell expression; levels affect liver/spleen/lung targeting	(42, 43)
PEG-Lipids	Hydrophilic polymer-lipid conjugates, alter size, circulation, and immune recognition	Extend circulation time, reduce opsonization, allow ligand targeting	PEG2000-DMG, PEG2000-DSG; varying PEG lengths influence ocular gene delivery and tissue-specific targeting	(44, 45)

### 3.2 Impact on delivery efficiency

Altering the composition of helper lipids in lipid nanoparticles using SORT (Selective Organ Targeting) strategy, which exploits the charge and type of helper lipids to direct LNPs to specific organs—liver, spleen, or lungs—without altering core nanoparticle structure, can influence the targeted delivery to different organs and tissues. It has been demonstrated that the charge of the helper lipids, not their polar groups, largely tells where the LNPs accumulate within the body. This finding was supported by either fully replacing an anionic or cationic lipid with a neutral phospholipid. For instance, adding cationic lipids causes accumulation in the lungs, whereas substituting an anionic zwitterionic helper lipid directs LNPs to the spleen. Furthermore, the percentage of cationic lipids has a major impact on biodistribution considering that lower concentrations of cationic lipids favor the liver or spleen, while higher concentrations target only the lungs. The apparent pKa of the LNPs is critical for their distribution, as it affects the formation of the protein corona on their surface. This protein corona interacts with tissue-specific receptors, guiding the LNPs to their target organs. A proposed three-stage mechanism involves the dissociation of PEGylated lipids from the LNP surface, allowing SORT-lipids to bind blood proteins, which then interact with receptors in specific tissues to achieve targeted delivery (46). Advanced techniques, such as using DNA barcode-labelled oligonucleotides, have enabled detailed *in vivo* analysis of LNP distribution, revealing factors like the length of hydrophobic tails, flexibility of sterol rings, and properties of PEG-lipids further enhance targeting efficiency (47).

To maintain membrane stability, LNPs frequently contain high cholesterol levels (up to 40 mol%), which also causes the liver to be the primary site of accumulation (43). While lowering the cholesterol content to 20% or 10% preserves the same initial physicochemical characteristics, stability is eventually jeopardized, leading to LNPs growing larger and losing mRNA. When given intravenously or intramuscularly, lower cholesterol levels cause LNP targeting to shift from the liver to the spleen and lungs. The key property of cholesterol that governs these effects is its rigid, planar sterol ring structure, which intercalates between lipid tails in the nanoparticle membrane, thereby modulating membrane packing, fluidity, and mechanical stability. Furthermore, without substantially changing biodistribution, modifying cholesterol with analogs such as 20*α*-hydroxycholesterol increases gene expression in particular liver cells. Researchers are creating cholesterol-free formulations or employing modified cholesterol derivatives, which maintain membrane stability while permitting precise organ targeting, to accomplish targeted delivery to organs other than the liver.

Because it encourages self-assembly, inhibits aggregation, and improves stability by encasing the particles in a protective shell, PEG is crucial for mRNA delivery in LNPs. Although too much PEG can over stabilize the membrane and prevent the fusion necessary for mRNA to enter cells from endosomes, this steric stabilization guarantees the homogeneity and durability of LNPs. Target protein expression has been demonstrated to increase when PEG content is decreased, suggesting that stability and functionality must be balanced. Furthermore, the size and distribution of LNPs can be precisely controlled by varying the molecular weight and molar percentage of PEG. PEG lipids based on phosphoglycerides are especially susceptible to these changes, which results in wider size distributions when high PEG concentrations are applied.

### 3.3 Modulation of immune response

The immune response triggered by LNPs themselves plays a critical role in the overall effectiveness and safety of mRNA vaccine formulations. As immunological activation from mRNA-LNP treatments can enhance the body's defenses but also lead to adverse effects like allergies and autoimmune diseases, it is crucial to balance boosting immunity with minimizing these reactions. To achieve this, strategies such as adjusting the composition and characteristics of LNPs, incorporating adjuvants, and controlling the injection route are essential for effectively modulating the immune response.

The formulation process makes it possible to precisely alter the four main lipids in LNPs; even small adjustments to lipid and PEG ratios, can have a big impact on the size and charge of the nanoparticles. Reducing the PEG ratio, for instance, can reduce the size of LNPs and increase their targeting efficiency to lymph nodes, which are essential for controlling the immune response. 30 nm LNPs were shown to target lymph nodes more successfully than larger (48). PEG can cause immunological reactions, like the generation of anti-PEG antibodies, which speed up the removal of nanoparticles from the bloodstream and lessen the effectiveness of repeated treatments (49).

Additionally, surface charge is essential to LNP operation. Although they may induce cytotoxicity and inflammation, positively charged liposomes can improve antigen delivery to antigen-presenting cells and boost immune responses (50). Ionizable lipids that stay neutral in the bloodstream are preferred by researchers to balance safety and efficacy. This reduces side effects while preserving the effectiveness of gene delivery. Neutrally charged LNPs efficiently reach draining lymph nodes without the negative effects of charged particles (51).

Adjuvants are essential for increasing the effectiveness of mRNA LNPs in cancer immunotherapy and vaccines by boosting the immune response. Adjuvants increase both innate and adaptive immunity by establishing a strong local immune environment at the injection site. In mouse tumor models, research has demonstrated that adding adjuvants such as PAM3CSK4, a TLR2 and TLR1 agonist, to LNPs enhances humoral immunity, cellular responses, tumor inhibition, and survival rates (52).

Since each route of administration interacts differently with the body's immune system, selecting the best one for mRNA LNPs is essential for maximizing immune responses. Several studies have demonstrated that intravenous administration significantly outperforms subcutaneous (SC) and intradermal routes in eliciting robust T-cell responses and antitumor efficacy. For instance, compared to SC or intramuscular injections, IV delivery of mRNA-lipoplexes produced greater numbers of antigen-specific CD8^+^ T cells and improved overall survival in tumor models. Furthermore, type 1 interferon (IFN) signaling impact on T-cell responses varies according to the mode of administration; when given intravenously, it boosts immunity, but when given subcutaneously, it suppresses it. Strong antibody and T-cell responses were elicited by ionizable LNPs, and intramuscular injections typically produced immunogenicity that was on par with or superior to that of intradermal and intranasal (IN) routes. But IN delivery by itself wasn't very successful (53, 54).

## 4 Designing optimized LNPs for platelet transfection

Platelets are fragments of megakaryocytes produced in the bone marrow that have a diameter of 1–3 µm (55). They play significant roles in the body namely homeostasis along with wound repair, inflammation and antimicrobial activities. They have certain advantages over other cells present in the peripheral blood like erythrocytes and leukocytes. On a daily basis, 2 × 10^11^ to 5 × 10^11^ platelets are produced with a lifespan between 7 and 10 days (56). Their higher production and shorter lifespan compared to erythrocytes and leukocytes enable a faster replenishment time. Platelets are discoid-shaped and have no nucleus, however, they do have mitochondria. This implies that they contain mitochondrial DNA but no nuclear DNA. Therefore, platelets do not possess the necessary transcriptional machinery to synthesize mRNA or regulate gene expression through transcriptional pathways. This results in an obstacle to delivering nucleic acids directly to platelets. Unlike nucleated cells, platelets rely on pre-existing mRNA and proteins stored during their production from megakaryocytes, limiting the effectiveness of traditional gene delivery methods (57). While platelets do contain translationally active mRNAs and the machinery for post-transcriptional modifications and translation, the lack of sustained synthesis pathways reduces the scope for therapeutic interventions using conventional strategies. Innovative approaches, such as leveraging LNPs for delivering functional RNA or exploring platelet-specific delivery mechanisms, are essential to address these challenges.

### 4.1 Challenges in platelet transfection

Platelet transfection with lipid nanoparticles presents several challenges. They primarily revolve around transfection efficiency, platelet activation, and compatibility with clinical practices. LNPs undoubtedly present a promising non-viral method for delivering mRNA to platelets, however, the aforementioned factors complicate their application. In one study, four different types of LNPs were analyzed: Cationic LNPs (cLNPs), Ionizable Cationic LNPs (icLNPs), LNPs Lacking Cationic Lipids (nLNPs) and the commercially available transfection reagent Lipofectamine (Lf) (58). Each formulation exhibited varying efficiencies in delivering mRNA to platelets. Cationic LNPs (cLNPs) showed higher transfection rates by delivering mRNA to the highest percentage of platelets. However, this in turn led to platelet activation impairing their function. Ionizable cationic LNPs (icLNPs) in contrast, delivered mRNA to fewer platelets without inducing activation, demonstrating a trade-off between efficiency and functionality. They did not impair platelet aggregation or spreading suggesting that it is possible to modify platelets genetically without compromising their essential functions (59). The study found another important point, that is, the mRNA delivered via both cLNPs and icLNPs remained stable in resting platelets. Only under specific conditions, the mRNA was released in platelet microparticles (MPs), indicating a potential mechanism for mRNA transfer to other cells (59).

Platelet Activation is another major challenge. The activation of platelets during transfection can compromise their hemostatic function. Hence, it is crucial to optimize LNP formulations to minimize activation while maximizing mRNA delivery. In one research by Leung et al. (60), it was demonstrated that human and rat platelets expressed exogenous proteins when treated with platelet-optimized mRNA-LNP. It was found that the expression of these proteins did not require platelet activation, nor did it correlate with it. Additionally, the genetically modified platelets retained their hemostatic function indicating that the modified platelets could still perform their primary role in blood clotting, which is critical for patient safety during transfusions. The platelets also functioned well *in vitro*; the transfused modified platelets were able to accumulate in areas of vascular damage in the rats with hemorrhagic shock. In another research by Strong et al. (61) it was found that a newly developed plasma-optimized mRNA-LNP can effectively transfect platelets directly in plasma and plasma supplemented with platelet additive solution. Such transfection method enhances scalability to both physiological and supraphysiological concentrations of platelets. It was also found that transfecting platelets with mRNA-LNP did not adversely affect their *in vitro* physiological functions increasing storage stability as well. These are all significant findings as they align with the current blood banking practices. This allows for a potential integration of genetically modified platelets into existing transfusion protocols.

### 4.2 Strategies for effective delivery

Effective delivery of RNA to platelets without activation or triggering an immune response is integral for the success of RNA-based therapeutics. In Table 2, we outline advanced strategies to overcome biological barriers and ensure the stability and efficiency of RNA delivery systems. Each LNP type serves a specific function in enhancing stability, minimizing immune detection, and promoting intracellular RNA delivery (62).

**Table 2:** Strategies for improved platelet transfection.

Strategy/component	Function/mechanism	Key details	Citation
Ionizable cationic lipids	mRNA complexation and cytosolic delivery via endosomal fusion	Protonated at low pH to enable endosomal fusion; neutral at blood pH to reduce toxicity	Ramachandran et al. (62); Kim et al. (63)
Helper lipids (e.g., lipidPEG)	Structural role, influence on fusogenicity	Lower fusogenicity; cleavable PEGylation used to address PEG-dilemma	Fang et al. (64)
Neutral phospholipids (DSPC & DPPC)	Influence biodistribution and release profile	DSPC (55°C) & DPPC (41°C) alter mRNA release from LNPs	Pilkington et al. (65)
Cholesterol	Enhances endosomal escape	Lowers LNP transition temp; facilitates phase transition for cytosolic release	Takahashi et al. (66); Allen & Cullis (67)
Zwitterionic phospholipids/Ionizable lipid ratio	Optimize binding and release of mRNA	Balance condensation and release of larger mRNA vs. siRNA	Liu et al. (68)
RNA chemical modifications	Targeted delivery via ligand bioconjugation	Improve transport efficiency and specificity	Kh. Abosalha et al. (69)
Formulation optimization (Design of Experiments)	Enhance targeted delivery, reduce liver accumulation	Enables delivery to tumor tissue and specific organs	Wu & Li (70)
Complementary peptides	Enhance organ-specific delivery	Improved mRNA delivery to heart and other organs	Wu & Li (70)
PEGylation (polyethylene glycol coating)	Immune evasion and circulation stability	Reduces immune detection; too much PEG reduces efficacy	Mariona Estapé Sentí (71)
Cationic polymers (lipofectamine)	Platelet RNA transfection	Optimal: 6-*μ*l lipofectamine, 400 pmoles siRNA in Tyrode's buffer; 14% transfection efficiency	Hong et al. (72)
Electroporation	Alternative delivery method	Less effective than optimized cationic lipid-mediated delivery	Hong et al. (72)

### 4.3 Optimization of LNP properties

Optimizing the properties of lipid nanoparticles is crucial to achieve efficient nucleic acid delivery, enhanced transfection, and minimal off-target effects. The primary determinant of LNP performance is the particle size, which influences biodistribution, cellular uptake, and immune response. Studies have shown that smaller LNPs, 100 nm in size, are effective in eliciting consistent and high antibody titers in animal models (73). While larger particles are relatively more organized with lamellar arrangements, they exhibit reduced surface polarity, which can impact cellular interactions and delivery efficiency. For an optimized LNP formulation, it must maintain high RNA encapsulation efficiency (>85%) with negligible aggregation, highlighting the robustness of size-controlled LNPs (73).

An optimal LNP formulation can be obtained by the combination of ionizable lipid, DSPC, cholesterol, and lipid-anchored polyethylene-glycol, with the ionizable lipid being most critical for mRNA expression. The preparation process for the LNP formulation typically involves dissolving lipids in ethanol and mixing with acidified aqueous mRNA solution to form particles through nanoprecipitation where an mRNA solution is rapidly mixed with a lipid solution in a controlled manner allowing the lipids precipitate out of the solvent and encapsulate the mRNA, forming stable nanoparticles. This process allows for the uniform distribution of mRNA within the lipid matrix, ensuring consistent particle size and encapsulation efficiency. Nanoprecipitation is favored due to its simplicity, scalability, and ability to produce LNPs with desirable properties for therapeutic applications. The particle size can be controlled by adjusting the aqueous-to-ethanol ratio Kimura et al. (74); He et al. (75) during the process of dissolving, with higher ethanol percentages (up to 50%) leading to larger particles. Moreover, flow rate also impacts size—lower total flow rates result in larger particles while maintaining consistent composition. The final formulation must undergo a buffer exchange to physiological conditions, concentration adjustment, and sterile filtration (76). This process not only allows the creation of LNPs with optimized properties but also maintains a high mRNA encapsulation efficiency. Currently, the traditional methods of ethanol injection and thin-film hydration are replaced by microfluidic mixing devices because they can produce homogenous lipid nanoparticles (LNPs) with narrower size distribution and higher mRNA encapsulation efficiency Roces et al. (77); Jahn et al. (78). The process begins by mixing the aqueous and ethanolic phases at pH 5.5, protonating ionizable lipids (pH < pKa) to bind and encapsulate mRNA. Gradually, bulk pH is increased until it reaches neutral via tangential flow filtration (TFF), causing ionizable lipids to become hydrophobic, driving vesicle fusion, and sequestering mRNA into the LNP interior (79).

In addition to size, lipid composition plays a pivotal role in tuning the functionality of LNPs as discussed in section 5.2. Ionizable lipids, for instance, enable efficient encapsulation of nucleic acids and promote endosomal escape, which is a critical step in cytoplasmic delivery. Machine learning approaches have been employed (more to be discussed in section 7) to identify optimal lipid ratios, such as 1:1 or 10:1 ionizable-to-helper lipids, that maximizes transfection efficiency across various cellular environments, including platelets Cheng et al. (80); Cheng et al. (78). Modifying the LNP surface, for instance, by the addition of polyethylene glycol (PEG) chains, can stabilize the particles and reduce immunogenicity. PEG lipids prevent excessive fusion, stabilizing particle size with a hydrophilic exterior, while neutral phospholipids like DSPC form a bilayer beneath the PEG layer (79). The formulation parameters such as lipid concentration, molar ratios, and ionizable lipid:mRNA ratios (N/P ratio) are very important to achieve optimal encapsulation and stability (81). Moderna demonstrated that the volumetric mixing ratio and total flow rate during microfluidic mixing significantly affect particle size Roces et al. (77); Hassett et al. (73). However, these benefits must be balanced with the potential for reduced cellular uptake due to steric hindrance.

Advanced targeting strategies also contribute to the optimization of LNP properties. Functionalizing LNPs with ligands such as transferrin or aptamers enhances their selectivity for specific cell receptors, which improves targeted delivery and minimizes off-target effects Song et al. (82); Yoo et al. (83). Furthermore, integrating immunosuppressive agents like corticosteroids into LNP formulations has shown the ability to mitigate inflammatory responses, particularly in therapeutic applications requiring repeated or prolonged dosing (84). All of these advancements collectively underscore the importance of tailoring LNP properties to the specific requirements of the therapeutic context, balancing delivery efficiency, safety, and immune compatibility (85–87).

## 5 Types of nucleic acid cargoes

Platelets have a natural ability to target sites of endothelial damage. Thus, modifying platelets can be advantageous in cell therapy. To increase protein expression in platelets, the cells need to be transfected, however, this brings a challenge. Platelets are anucleate cells which indicates that they do not possess the traditional mechanism of protein synthesis like nucleated cells (88). Instead, RNA-based agents need to be delivered into the cytoplasm of the platelet. But certain factors need to be maintained in order to achieve that; RNA must reach the cytoplasm without triggering any immune response and activation of platelets so multiple signaling pathways must be avoided Karikó et al. (89); Fröhlich (57). Therefore, finding an appropriate transfecting agent is important. As discussed in the earlier sections, lipid nanoparticles served as a suitable transfecting agent because they protect the cargo from degradation and can be easily taken up by cells through endocytosis.

Mostly, mRNA, small-interfering RNA, and microRNA (miRNA) have been delivered to platelets (59). Delivering nucleic acids to platelets has several advantages over directly delivering exogenous proteins (60). For instance, mRNA serves as one common template for ribosomes present inside the platelets to be translated into a wide range of different proteins. Therefore, depending on the therapeutic need, the sequence of the nucleic acid payload can be changed to achieve different proteins. Apart from flexibility in protein production, a higher concentration of protein can be produced because of multiple rounds of translation. In Table 3, we have outlined the different nucleic acid cargos that can be delivered to platelets and their therapeutic potential.

**Table 3:** Outcomes of nucleic acids delivery to platelets.

Delivery type	Study	Method/carrier	Target/content	Outcome	Key insight
mRNA	Chan et al. 2015 (85)	RNA-synthesizing nanoliposomes	Exogenous DNA/mRNA	59-fold increase in mRNA after irradiation; no protein translation in platelets	Successful transcription, but delivery ≠ expression
mRNA	Novakowski et al. 2019 (59)	4 types of LNPs: Lf, nLNPs, cLNPs, icLNPs	GFP mRNA	icLNPs showed minimal platelet activation	Ionizable cationic lipids are safer for platelets
mRNA	Lu et al. 2023 (71)	Novel ionizable LNPs (double ethanolamine head group)	mRNA	Enhanced binding, stability, and protein expression	Amine head group chemistry affects efficiency
siRNA	Hong et al. 2011 (72)	Lipofectamine 2000	siGAPDH	33% decrease in GAPDH mRNA; scrambled siRNA = 26% reduction	Platelets can be transfected; controls are critical
siRNA	Yousefi et al. 2014 (90)	Angiplex (lipid-based) vs. pHPMA-MPPM (polymer-based)	siVEGFR-2	Minimal platelet/coagulation activation with Angiplex	Lipid-based carriers are more hemocompatible
miRNA	Lazar et al. 2020 (91)	Gymnosis	miR-223-3p	↓ SEPTIN2 translation; ↓ microparticle formation	Platelets can internalize miRNA via gymnosis
miRNA	Cepparulo et al. 2024 (92)	Transferrin-conjugated LNPs	anti-miR-103/107	Crossed BBB, reduced ischemic volume, low toxicity	Targeting ligands may outweigh lipid content

## 6 Intelligence as a transformative tool in nanoparticle design

**Figure 2:**
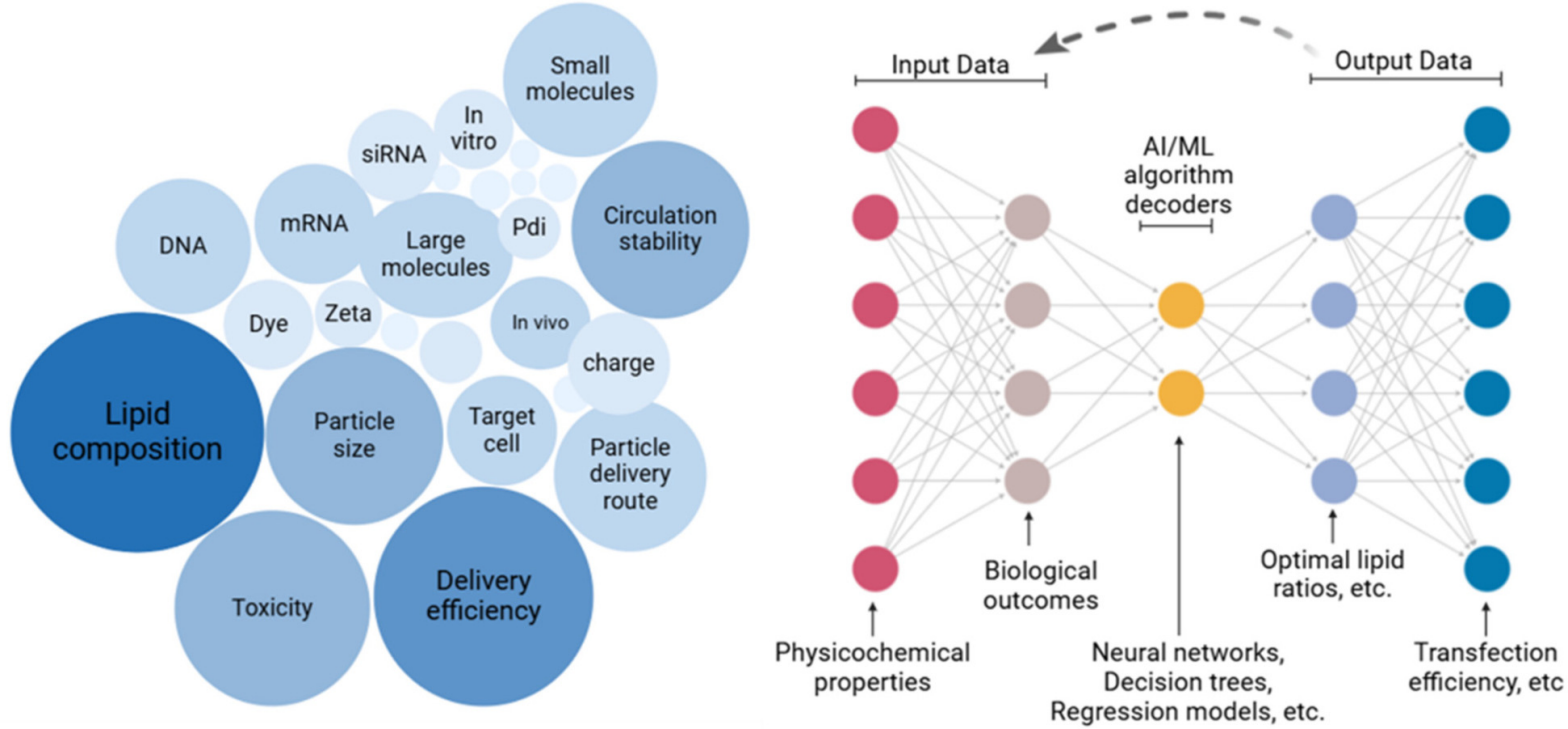
AI-Driven predictive modeling in LNP design. A large dataset represented by a cluster of nodes with labels, Input layer: Data types fed into an AI model (e.g., physicochemical properties, biological outcomes), Middle layer: Represent AI/ML algorithms with icons like neural networks, regression models, or decision trees, Output layer: Predictive outcomes (e.g., optimal lipid ratios, encapsulation efficiency), and Feedback loop: Dashed arrows linking AI outputs back to experimental design.

Artificial Intelligence refers to the capability of machines to employ advanced algorithms trained on existing data to make predictions, handle unexpected situations effectively, and achieve specific objectives. This is primarily executed through computer programs. A significant branch of AI is Machine Learning (ML), which focuses on utilizing statistical methods to learn from data and perform tasks without explicit programming. Among various AI approaches, ML has gained prominence, especially with recent advancements in deep learning techniques. Lipid nanoparticles are vital delivery systems for mRNA therapeutics, with applications ranging from vaccines to treatments for genetic diseases and cancer. Clinically approved LNPs consist of four key components: ionizable cationic lipids for mRNA encapsulation and endosomal release; cholesterol for stability and circulation; helper phospholipids for nanoparticle stability and delivery efficiency; and PEGylated lipids to prevent aggregation. Biodistribution, primarily influenced by lipid composition, administration route, and particle size determines the accumulation of LNPs in specific organs, often favoring the liver and spleen due to their sinusoidal endothelium. To enhance targeted delivery, researchers are exploring strategies such as altering lipid types and ratios, replacing helper lipids or PEGylated lipids, adjusting cholesterol content, and employing novel ionizable lipids with specific structural features. Computational techniques like machine learning are increasingly used to predict LNP properties and expedite formulation optimization. See Figure 2. By tailoring lipid composition, scientists aim to improve the specificity, efficacy, and safety of mRNA-based therapies for diverse applications (43).

Extracellular vesicles (EVs), particularly exosomes, have a natural ability to transport molecules between cells which makes them attractive candidates for delivering therapeutic agents. However, EVs derived from the same studies can exhibit variations in size, molecular content, and surface markers; This heterogeneity complicates the prediction and control of their targeting specificity and therapeutic effectiveness. AI has emerged as a potential solution to overcome these limitations and unlock the full therapeutic potential of EVs. AI algorithms can identify optimal targeting ligands or surface modifications that improve EV binding to specific cell types. Furthermore, AI-driven multi-omics analysis of EVs can provide valuable insights into disease mechanisms and potential therapeutic targets. EVs carry a diverse array of biomolecules including DNA, RNA, proteins, and lipids that reflect the state of their originating cells. By analyzing these molecular profiles using AI algorithms, researchers can identify disease-specific signatures and potential biomarkers for diagnosis, prognosis, and treatment monitoring. This information can then be used to develop personalized EV-based therapies tailored to an individual patient's molecular profile. By addressing challenges associated with EV heterogeneity, standardization, and formulation, AI can accelerate the development of safe, effective, and targeted EV-based therapies for a wide range of diseases (93).

### 6.1 Core machine learning methodologies in nanoparticle research

**Figure 3:**
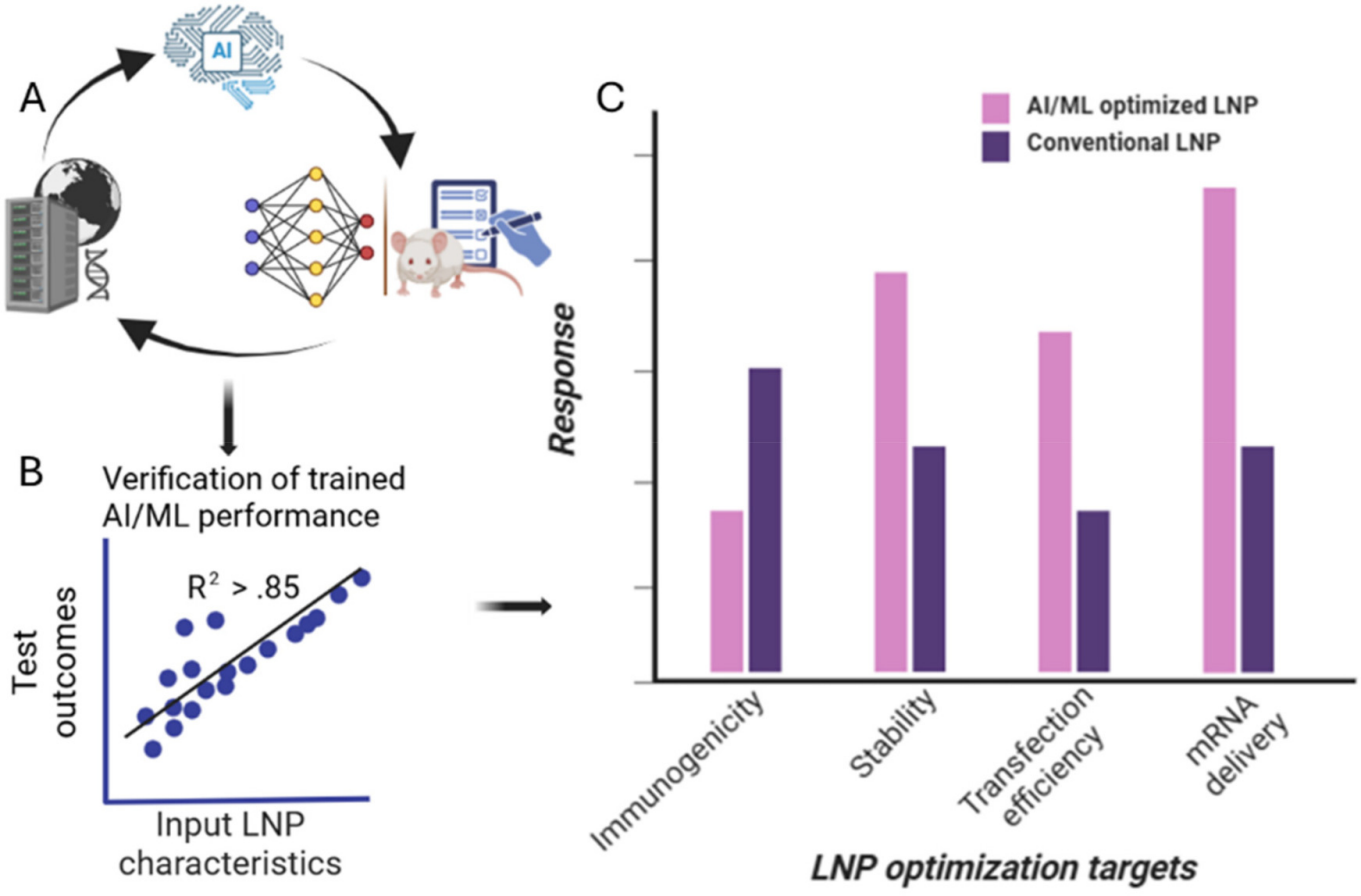
Case study of AI application in LNP formulation. **(A)** Large datasets are first used to train AI/ML algorithm where its outputs are compared to experimental data to gauge predictive power. Process is iterated to enhance usefulness of algorithm. **(B)** AI/ML algorithm is then trained to reach high correlation before **(C)** algorithm validation is performed using comparative chart development where traditional formulation outcomes of average LNP properties including immune response, transfection efficiency, stability and mRNA delivery are compared to AI-optimized formulation showing higher-performing metrics for the same parameters.

The field of machine learning algorithms is broad and selecting the best one to use can be a daunting undertaking. Among the algorithms currently in use, a dozen of the well-known ones is described in Table 4 below. What is common with these algorithms is the goal to link inputs and predictions swiftly and economically with low error association. See Figure 3. However, how the link is performed, processing speed, resource cost, and prediction accuracy can differ for each algorithm. Furthermore, the areas where they are commonly applied, input data types and processing format can be a good guide for which algorithm to explore. In the following section, machine learning algorithms for nanoparticle optimization are explored.

**Table 4:** Most used machine learning algorithms.

Algorithm	Description	Application areas
Bayesian Optimization (BO) (94).	-Bayesian optimization is a sequential approach to optimizing black-box functions without assuming any specific functional form. - Works by treating the objective function as a random process and starting with a prior assumption about its behavior. As function evaluations are performed, this prior is updated with new data to form a posterior distribution, which better represents the function.	This function strikes a balance between exploring unknown areas and focusing on promising regions to find the best results efficiently
Deep Neural Network (DNN) (95).	-A Deep Neural Network (DNN) is a type of computer model designed to mimic how the human brain processes information -What makes DNNs powerful is their ability to handle complex problems, like translating languages, diagnosing diseases, or predicting stock prices. They learn by adjusting their internal connections through a process called training, which involves showing the network lots of examples and comparing its predictions to the correct answers	-While DNNs are incredibly versatile, they can sometimes behave like a black box, meaning it's hard to fully understand how they make certain decisions. -Widely used in areas like self-driving cars, voice assistants, and image recognition
LightGBM (96).	- LightGBM, or Light Gradient Boosting Machine, is a machine learning algorithm designed for speed and efficiency, particularly when working with large datasets. It is widely used for tasks like prediction, classification, and ranking, where high performance is essential. The algorithm works by building a series of decision trees -This approach allows it to focus on areas where the model performs poorly, improving accuracy while reducing computation time.	The algorithm is commonly applied in fields such as finance, e-commerce, and healthcare for tasks like fraud detection, recommendation systems, and customer behavior analysis.
XGBoost (97).	XGBoost, short for Extreme Gradient Boosting, is a powerful and efficient machine learning algorithm used for tasks like classification, regression, and ranking -XGBoost builds decision trees sequentially, with each new tree focusing on correcting the errors made by the previous ones. This process continues until the model achieves the desired level of accuracy	Widely used in industries like finance, healthcare, and marketing for applications such as credit scoring, disease prediction, and customer segmentation.
Support Vector Machines (SVM) (98).	-Support Vector Machines (SVM) is a machine learning algorithm commonly used for tasks like classification and regression. It is particularly effective in situations where the goal is to separate data into distinct categories or predict numerical outcomes - The algorithm is known for its ability to handle high-dimensional data and perform well with a small number of samples	SVM is widely applied in areas like text classification, image recognition, and bioinformatics for tasks such as spam filtering, face detection, and gene classification
Random Forest Regression (99).	-Random Forest Regression is a machine learning algorithm used for predicting numerical values by combining the results of multiple decision trees - Each tree provides a prediction, and the final result is calculated as the average of all these predictions. This approach helps minimize overfitting, as no single tree dominates the model, and improves performance on complex datasets.	Widely used in fields like finance, healthcare, and environmental science for applications such as stock price prediction, medical diagnosis, and climate modeling
Gaussian Process (GP) (100).	Gaussian Process (GP) is a machine learning method often used for regression and probabilistic modeling - When making predictions, GP uses the data it has seen to calculate a distribution of possible outputs for new points. This allows it to provide both a predicted value and a measure of uncertainty, represented as a confidence interval.	Useful for tasks like optimization and risk assessment, where understanding uncertainty is critical.
Self-Validated Ensemble Model (SVEM) (101).	A Self-Validated Ensemble Model is a machine learning approach that combines predictions from multiple models to improve accuracy and reliability while incorporating a built-in validation mechanism. -By combining the insights from multiple models and incorporating self-validation, these ensembles provide robust predictions with a high degree of reliability and generalizability, even when dealing with noisy or complex datasets.	Useful in situations where accuracy is critical, such as healthcare diagnostics, financial forecasting, and risk analysis
Artificial Neural Networks (ANN) (102).	Artificial Neural Networks (ANNs) are a type of machine learning model inspired by the structure and function of the human brain. They consist of layers of interconnected nodes, called neurons, that process and learn from data by identifying patterns and relationships. - They are particularly effective for problems where the underlying patterns are complex and not easily captured by traditional methods	Used for tasks like image and speech recognition, language translation, and financial forecasting
Principal Component Analysis (PCA) (103).	Principal Component Analysis (PCA) is a technique used in data analysis and machine learning to reduce the number of variables in a dataset while retaining its most important information - This technique is particularly useful for visualizing high-dimensional data, speeding up machine learning algorithms, and eliminating redundant or irrelevant features	PCA is widely used in fields like image compression, finance, and genetics for tasks such as pattern recognition, anomaly detection, and feature extraction.
Partial Least Squares (PLS) (104).	Partial Least Squares (PLS) is a statistical and machine learning method used to model relationships between input variables (predictors) and output variables (responses). It is particularly useful when the predictors are highly correlated or when there are more predictors than observations, which can make traditional regression techniques ineffective. - Unlike Principal Component Analysis (PCA), which focuses only on the predictors, PLS takes the response variables into account when identifying these components.	This method is widely used in fields like , bioinformatics, and social sciences for applications such as spectroscopic data analysis, gene expression studies, and customer preference modeling.
Reinforcement Learning (RL) (105).	Reinforcement Learning (RL) is a type of machine learning where an agent learns to make decisions by interacting with an environment. Instead of being given explicit instructions, the agent learns through trial and error, aiming to maximize a long-term reward - One of RL's key strengths is its ability to learn from sparse feedback and adapt to dynamic situations. However, it can require significant computational resources and many iterations to converge on a good solution, especially in environments with high complexity or delayed rewards.	Particularly effective in tasks where the goal is to find an optimal sequence of actions, such as game playing, robotics, autonomous driving, and resource management.

### 6.2 Algorithmic strategies for lipid nanoparticle optimization

ML algorithms have paved the way to predict the best strategies for manufacturing or synthesis of nanoparticles. Mekki-Berrada et al. (2021) developed a two-step machine learning framework combining Bayesian Optimization and a Deep Neural Network to optimize silver nanoparticle synthesis with tailored optical properties. The approach, tested on 120 experimental settings, accelerates synthesis by refining conditions and provides insights into how chemical composition affects optical behavior (43, 106) used the LightGBM algorithm to optimize LNP formulations by analyzing 325 formulations, achieving strong predictive performance (R^2^ > 0.87) and identifying structural features of ionizable lipids linked to efficacy. Their study, validated experimentally and supported by molecular dynamics simulations, highlights advancements in machine learning for LNP design, enabling efficient mRNA vaccine development and revealing electrostatic interactions as key to mRNA encapsulation.

In a different study, researchers created 24 mRNA-LNP formulations using an I-optimal design, optimizing material attributes and processing conditions to enhance critical quality attributes like particle size, Zeta potential, and encapsulation efficiency (107). By leveraging machine learning tools, including a self-validated ensemble model (SVEM) with over 97% accuracy, the study identified key factors influencing mRNA-LNP quality and optimized manufacturing conditions, demonstrating the potential of AI in vaccine development. A combinatorial artificial neural network-design of experiment (ANN-DOE) model optimized mRNA-LNP bioprocessing by analyzing factors like lipid type, lipid-to-cholesterol ratio, N/P ratio, and flow rates (102). This method outperformed other machine learning models in predicting critical attributes (e.g., particle size, Zeta potential, and encapsulation efficiency), providing a cost-effective strategy for improving LNP production for gene therapies and nucleic acid treatments.

Some other studies suggest that traditional optimization methods often struggle with the complexity and variability of biological systems, where ML excels in identifying patterns and enabling predictive modeling (108). Techniques like SVM, PCA, and reinforcement learning have been applied to optimize processes, predict yields, and enhance scalability, though challenges like data quality and expertise remain barriers to integration. In their article Van der Meel et al. (109) discuss integrating ML with high-throughput synthesis to accelerate the discovery of ionizable lipids for mRNA delivery, overcoming the limitations of traditional methods. This ML-guided approach streamlines lipid identification, enhances delivery performance, and supports rapid development of effective LNPs for mRNA-based vaccines and therapies.

In a groundbreaking study (110), researchers studied how variations in LNP composition affect mRNA delivery and immune responses, analyzing 213 formulations using random forest regression models. Their findings highlighted the importance of phenol groups and hydroxyl-functionalized ionizable lipids for efficient mRNA encapsulation, enhanced expression, and robust immune responses, providing insights for designing potent and safe mRNA therapeutics.

In a separate study (111) researchers used machine learning to optimize LNP manufacturing for mRNA delivery, focusing on particle size and quality control emphasized by the FDA. By combining XGBoost and Bayesian optimization, they identified ethanol concentration and pH as key factors, achieving precise, scalable, and efficient LNP production for early-stage formulation development.

Researchers developed fucoidan/polyethyleneimine (PEI) nanoparticles for sorafenib delivery in cancer therapy, optimizing formulation parameters using machine learning and a DoE-ANN approach (112). The resulting nanoparticles demonstrated controlled drug release, cancer site retention, and synergistic anticancer effects, highlighting the potential of these technologies for targeted drug delivery.

### 6.3 Application examples of predictive modeling of lipid nanoparticle properties and biological behavior

One study (113) presents a ML approach to predict the transfection efficiency of LNPs used for mRNA delivery. The researchers curated a dataset of 622 LNPs from existing studies, categorizing them into those with satisfying and unsatisfying transfection efficiency based on expert knowledge. The ML model utilizes molecular representation learning techniques to encode the chemical structures of the LNPs’ four components. Two approaches were employed for molecular representation: “expert fingerprints,” which extract features based on chemical domain knowledge, and “neural fingerprints,” generated using graph neural networks (GNNs). These representations were combined with information about component ratios and fed into various classification models, including SVM, Random Forest, XGBoost, and MLP. The study found that models trained with “expert fingerprints” outperformed those using “neural fingerprints” in predicting LNP transfection efficiency. This suggests that in this specific application, domain knowledge-based feature extraction is more effective than GNN-based representation learning. The best performing model, a multilayer perception trained with “expert fingerprints,” achieved a remarkable 98% accuracy on the test set. This result demonstrates the potential of ML to accelerate the development of LNPs for mRNA delivery by predicting their functionality based on their chemical structure, thereby prioritizing promising candidates for experimental validation. This approach resonates with other studies in our conversation history, where ML models trained on experimental datasets successfully predicted the properties of different drug delivery systems, including solid lipid nanoparticles, polymeric microparticles, and even extracellular vesicles.

Another study (79) focuses on the development of LNPs for delivering siRNA) to silence disease-causing genes in hepatocytes. The study highlights the importance of ionizable cationic lipids in LNP systems, as they play a critical role in siRNA entrapment and intracellular delivery. The researchers used microfluidic mixing to prepare LNP-siRNA systems containing four lipid components: hydrogenated soy phosphatidylcholine, cholesterol, PEG-lipid, and 1,2-dioleoyl-3-dimethylammonium propane. A design of experiments approach was used to systematically investigate the effect of various preparation parameters, including lipid concentration, flow rate ratio (FRR), and total flow rate. The results indicated that lipid concentration and FRR significantly impacted the particle size and polydispersity index (PDI), while siRNA encapsulation remained consistently high around 90%. The study also investigated the impact of the dialysis process, used to remove ethanol and adjust the pH of the LNP-siRNA systems. Interestingly, a decrease in PDI and an increase in particle size were observed after dialysis, particularly for systems prepared with a low FRR (more ethanol). This observation was attributed to the neutralization of the ionizable lipid DODAP during dialysis, leading to reduced intervesicle repulsion and subsequent particle fusion. Moreover, the presence of siRNA was found to influence the particle size and PDI, likely by limiting lipid rearrangement due to complex formation between siRNA and the ionizable cationic lipid. The study concludes that careful control of preparation parameters, especially lipid concentration and FRR, is crucial for achieving desired LNP-siRNA properties for effective siRNA delivery.

**Figure 4:**
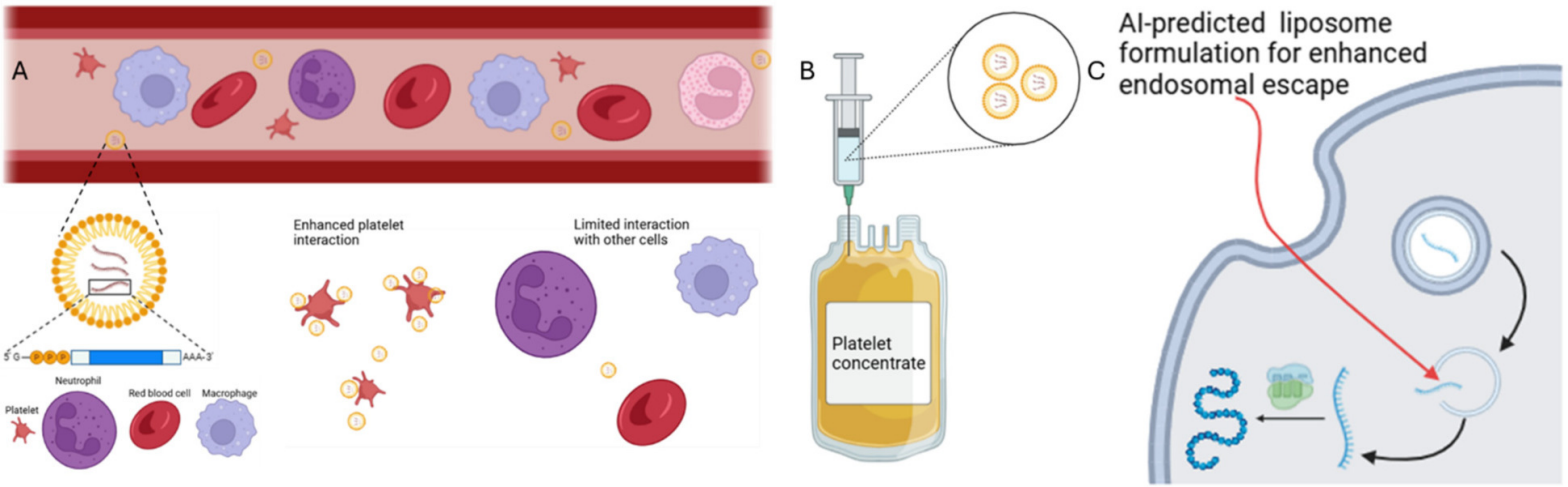
mRNA delivery by LNPs to platelets by intravascular administration or by injection into platelet concentrate. IV administration of LNP is illustrated in panel **(A)** where AI-predicted lipid composition enhances platelet cell interaction over other cell types. The injection of these LNPs into platelet concentrates, panel **(B)**, offer unique advantages such as elimination of off-target effects and high concentration of the targeted cell. In both targeting approaches, AI-predicted lipid formulation can enhance endosomal escape to potentiate phenotypical expressions **(C)**.

This scientific article (114) investigates the impact of ionization and structural properties of mRNA LNPs on their effectiveness in delivering mRNA for intramuscular (IM) and intravascular (IV) administration. The central component influencing LNPs' delivery efficiency is the pKa value, which represents the acidity of the nanoparticle. This study found that the pKa of an LNP is generally 2–3 units lower than the pKa of the ionizable lipid it contains. This difference stems from variations in proton solvation energy between the LNP and the surrounding aqueous environment. The researchers employed diverse methodologies, including theoretical calculations, Nuclear Magnetic Resonance spectroscopy, fluorescent dye binding assays, and electrophoretic mobility measurements, to thoroughly examine the protonation behavior of both ionizable lipids and LNPs. They discovered that LNPs with a more negative charge tend to exhibit increased off-target expression of mRNA in the liver after IM administration, which is an undesirable effect. This off-target expression could potentially be mitigated by optimizing the design of ionizable lipids and the LNPs themselves. Furthermore, the study revealed that lowering the ratio of lipid to mRNA in LNPs resulted in larger and more negatively charged particles. These larger LNPs demonstrated superior potency, likely due to enhanced protonation within the acidic environment of endosomes, cellular compartments involved in the uptake and processing of external materials. The researchers also discovered a strong correlation between LNP potency *in vitro* and *in vivo* for IM injections, suggesting the predictability of real-world performance based on laboratory experiments. However, this correlation was not observed for IV administration, possibly due to differences in charge-mediated interactions with biological molecules, such as extracellular matrix components for IM and Apolipoprotein E for IV delivery. Insights gained from this study, including the understanding of LNP pKa, the role of LNP charge, and the impact of lipid-to-mRNA ratio, offer valuable guidance for the rational design of more potent and targeted mRNA LNPs for platelet applications and others, including vaccines and therapeutics. See Figure 4.

### 6.4 Simulation of nanoparticle-cell interactions

Simulation of nanoparticle-cell interactions plays a critical role in understanding the complex dynamics between nanoparticles and biological systems. By providing insights into how nanoparticles adhere to, penetrate, and affect cell membranes, these simulations help predict their behavior *in vivo*, allowing for the optimization of their design for targeted drug delivery, imaging, and therapeutic applications. Moreover, simulations can reveal potential toxicological effects, interactions with biomolecules, and the overall impact on cellular function, thereby guiding the development of safer and more efficient nanomedicines.

In a related work, atomistic and coarse-grained simulations are compared to assess how nanoparticles interact with biological systems, focusing on two coarse-grained models, POL-MARTINI and BMW-MARTINI. Both models showed qualitative agreement with atomistic simulations regarding surface properties of amine-functionalized gold nanoparticles, but with differences in charge oscillation and salt ion resolution (115). The BMW-MARTINI model closely resembled atomistic interfacial properties, while POL-MARTINI underestimated nanoparticle binding to Cl− and water. The models also differed in predicting nanoparticle binding to lipid membranes, with POL-MARTINI showing no affinity for either zwitterionic or anionic bilayers, while BMW-MARTINI overestimated the cationic nanoparticle's affinity to zwitterionic bilayers. The study emphasizes the importance of charge and water distributions at the particle-water interface in determining nanoparticle interactions with other molecules. Additionally, the role of nanoparticles in affecting cell mechanics, particularly cell adhesion, cytoskeletal organization, and stiffness, which are critical for various cellular functions has been reported (116). Nanoparticles can disrupt cell adhesion and cytoskeletal components, leading to compromised tissue integrity and abnormal cell migration. The impact of nanoparticles on cell stiffness is complex, with varying effects depending on particle properties. The study also highlights how nanoparticles influence cell motility, with smaller nanoparticles generally promoting migration. These findings underline the need for further research into the relationship between nanoparticles and cell mechanics to develop safer, more effective nanomaterials for biomedical applications. Additionally, a study on lipid-based liquid crystalline nanoparticles, including cubosomes and hexosomes, found that both nanoparticles exhibited similar cellular interactions despite expected differences (117). Researchers suggest that nanoparticle transformation upon cell contact influences their interaction with cells, demonstrating the importance of advanced analytical techniques for accurately interpreting nanoparticle behavior in biological environments.

## 7 Challenges and future perspectives

Significant advancements in nucleic acid delivery have been made, yet key challenges remain. While lipid nanoparticles move the needle on gene therapy and mRNA vaccines formulations, enhanced delivery efficiency is still a hurdle. Stability is a major issue, as LNPs must navigate the body without degrading. Targeting the right cells is another challenge, as off-target effects can cause unintended consequences. Additionally, the immune system may attack LNPs, reducing their effectiveness. Overcoming these barriers through improved formulation and targeting strategies is crucial for developing more effective treatments. See Table 5.

**Table 5:** Strategies for enhancing nucleic acid delivery via lipid nanoparticles.

Strategy	Examples
Tailoring LNP composition	*Ionizable lipids (DLin-MC3-DMA used in mRNA vaccines):* (118) The development of new ionizable lipids optimized for endosomal escape and reduced toxicity can enhance nucleic acid release within cells. *Cholesterol Analogues:* (119) Modified cholesterol enhances membrane fusion and stability of LNPs. *PEG-Lipids with Tunable Properties:* Using PEG-lipids that detach under physiological conditions improves circulation time without hindering cellular uptake.
Enhanced targeting strategies	*Ligand-Functionalized LNPs* (120) Attaching targeting ligands (e.g., aptamers, peptides, antibodies) to LNPs enables specific delivery to tissues or cells. Example: ASGR1-targeting ligands for hepatocyte-specific delivery. *Surface Engineering:* (87) Incorporating molecules like transferrin or folate enhances receptor-mediated endocytosis. *pH-Responsive Lipids:* Designing lipids that respond to the acidic tumor or endosomal environments can boost targeted delivery.
Improving endosomal escape	*pH-Sensitive Components:* (121) Inclusion of fusogenic lipids or peptides that destabilize the endosomal membrane in acidic environments. *Proton Sponge Effect:* (122) Using polymers or ionizable lipids that cause osmotic swelling and rupture of the endosome.
Optimizing nucleic acid encapsulation	*Polyanion-Lipid Complexes:* (67 ) Improved methods for compacting nucleic acids with polyanions prior to encapsulation, ensuring higher stability and payload efficiency. *Charge Ratio Fine-Tuning:* (123) Optimizing the charge ratio of lipids to nucleic acids enhances encapsulation and reduces aggregation.
Controlled release mechanisms	*Stimuli-Responsive LNPs:* (124) Systems that release nucleic acids upon exposure to stimuli like light, enzymes, or temperature. E*nzyme-Cleavable Linkers:* (125) Linkers that degrade in specific cellular environments (e.g., cancerous tissues) ensure precise nucleic acid release.
Combination therapies	*Co-Delivery of Modulators:* (126) Delivering nucleic acids alongside small molecules (e.g., immune suppressors, endosomal escape enhancers) to synergize therapeutic outcomes. *Dual Payloads:* (127) Co-encapsulation of different nucleic acids (e.g., siRNA and mRNA) for combinatorial effects.
Advances in manufacturing and scalability	*Microfluidics:* (128) High-throughput, reproducible LNP formulation using microfluidic systems for precise control over size and encapsulation efficiency. *Nanoparticle Tuning with AI:* (129) Using machine learning to predict optimal lipid compositions for specific delivery challenges.
Reducing immunogenicity	*Stealth Modifications:* (130) Further PEGylation optimization or use of zwitterionic coatings to reduce immune recognition. *Protein Corona Avoidance:* (131) Designing surfaces that minimize protein adsorption, thus improving circulation time and targeting accuracy.
Exploring alternative lipid architectures	Hybrid LNPs (132): Combining lipids with polymers, peptides, or dendrimers for enhanced delivery properties. *Lipid-Derived Prodrugs:* (133) Designing lipids that actively participate in therapeutic activity post-delivery.

The stability of LNPs has been investigated using different modifications approaches, from physical reinforcements for protection from vesicle damage to chemically focused formulation profiles for reduction of undesired biological interactions. Cholesterol, now viewed as a beneficial formulation component for enhancing the stability of LNPs, was investigated early in the nineties by Huang Leaf's group (Huang et al) (134). In their study, 3β[N-(N',N'-dimethylaminoethane)-carbamoyl]cholesterol, or DC-Chol, was reportedly chosen for its biocompatibility and the stability it imparts to lipid membranes. Mammalian cells transfection experiments leading to an observed transfection activity of up to two- to four-fold greater chloramphenicol acetyltransferase expression (CAT assay) and a four-fold reduction in cytotoxicity vs. Lipofectin in some cell lines was supportive evidence for the choice of cholesterol as LNP formulation material.

Cholesterol type, molar ratio, and their impact on biocompatibility and LNP transfection efficiency have been studied. Patel and Sahay (42), found that incorporating C-24 alkyl phytosterols enhances gene transfection, requiring specific alkyl tail length, sterol ring flexibility, and -OH group polarity. Nguyen and Szoska (135), highlighted LNP stability dependence on composition, as liposome components can exchange with lipoproteins. Proper molar ratios and avoiding excess polymer chains or liposome materials help reduce material shedding, preventing particle destabilization, altering biodistribution, or increasing clearance *in vivo* (136, 137). Incorporating lipid PEG in LNPs enhances biocompatibility and protects against degradation *in vivo* (138–142). PEG is valued for its versatility, renal clearance, and ease of application (138). Liposomes can be PEGylated by adsorption or covalent attachment (139). Kim et al. (18) found PEG-modified lipoplexes achieve higher transfection rates in serum-rich conditions. PEG creates a steric shield, reducing macrophage uptake and aggregation, improving drug bioavailability (139–141). However, PEGylated LNPs may require immunogenicity monitoring (142) and can hinder endocytosis, depending on PEG proportion and functional groups (143).

Off-target LNP accumulation in the liver and macrophage clearance hinder targeted delivery, prompting research into surface modifications with ligands and tissue-specific genetic cargo. Targeted delivery relies on cell surface receptors and protein expression in response to disease. While known targets guide LNP design, identifying novel protein targets requires combinatorial peptide libraries to screen ligand binding affinity. Structural analysis using protein databases and computational tools like POCASA or Fpocket aids rational peptide design. Phage display libraries can then identify peptides that bind the target protein. PEG lipids and LNP surface modifications further improve targeting, enhancing gene delivery and safety. Ensuring the long-term safety of gene delivery requires extended patient monitoring and a rigorous approval process. The widespread use of LNP-mRNA vaccines began in 2019, making long-term effects uncertain. The COVID-19 mRNA vaccines used ionizable cationic lipid, cholesterol, DSPC, and PEG-lipid (144), necessitating further study of each component, especially in marketed LNP drugs like Patisiran (Onpattro®), BNT162b2 (Comirnaty®), and mRNA-1273 (Spikevax®) (145). PEG can induce anti-PEG antibodies, forming antigen-antibody complexes that accelerate drug clearance by macrophages, reducing biodistribution and limiting efficacy (146–148).

Clinical studies on PEG antibodies are limited and inconclusive due to small sample sizes (149, 150), variability in pre-existing antibodies (149, 151), demographic factors, sampling deviations, and mixed LNP drug use (151). Wang et al. (152) found PEGylated LNPs in mice induced a dose-dependent immune memory, accelerating anti-PEG IgM/IgG response and clearance upon re-injection in rats. While FDA approval and COVID-19 emergency authorization have benefits, further research on PEG, cholesterol, and other formulation components can refine LNP drug delivery and clinical guidelines. Addressing stability, off-target effects, and immunogenicity in LNPs offers opportunities to optimize formulations and advance clinical applications.

Ongoing clinical trials highlight LNPs’ broad potential in nucleic acid delivery. mRNA vaccines are being tested for personalized cancer immunotherapy, such as advanced melanoma, where LNPs deliver tumor-specific neoantigens to stimulate immune responses alongside checkpoint inhibitors like pembrolizumab (153). In infectious diseases, an influenza vaccine trial is evaluating two doses of the DCVC H1 HA mRNA vaccine administered 28 days apart (154). Another trial assesses an investigational mRNA vaccine for preventing lower respiratory tract infections caused by RSV and/or human metapneumovirus in older adults (155).

For genetic disorders, LNP-based mRNA therapies aim to correct conditions like ornithine transcarbamylase (OTC) deficiency. ARCT-810, an LNP-formulated OTC mRNA, is being tested for safety and pharmacodynamics in adolescents and adults (156). These trials underscore LNPs' versatility in stabilizing and targeting nucleic acid therapies while addressing immunogenicity and off-target effects. Gene delivery to platelets offers promises for improving storage, transfusions, and disease treatment. See Figure 4. Though anucleate, platelets can be genetically modified via nanoparticles. Introducing anti-apoptotic genes like Bcl-xL may extend platelet shelf-life, while engineered platelets could enhance transfusion efficacy by carrying hemostatic agents or surface antigens to reduce alloimmunization risks. Modified platelets expressing Factors VIII or IX could improve hemophilia treatment, while those carrying thrombopoietin may aid thrombocytopenia. Engineered platelets could also release anti-inflammatory or anti-thrombotic factors for cardiovascular therapies.

A key challenge in antithrombotic therapy is preventing pathological thrombosis without impairing normal hemostasis, which can lead to bleeding complications. The emerging concept is that platelets are very heterogeneous. Our lab and others have demonstrated that there are subpopulations of platelets expressing different functional markers with distinct phenotypes that are likely most contributory to disease (157–159). Pathogenic platelet subpopulations, characterized by hyperactivation, increased procoagulant activity, and/or dysfunction, may drive thrombotic processes (160–164). Targeting the pathogenic platelet populations while sparing healthy inactive platelets may be key to reducing thrombosis risk while preserving normal hemostasis. One approach is to use nanoparticle delivery of drugs that recognize and bind selectively to markers expressed predominantly on highly active and dysfunctional platelets. Platelet activation markers, such as P-selectin, activated GPIIb/IIIa, and CD63, may be targeted by specific antibodies or small molecules that can modify or eliminate these pathogenic subsets. Another approach relies on the variations in membrane composition of distinct platelet populations. In particular, procoagulant and apoptotic platelets, contain high levels of negatively charged phosphatidylserine on the outer surface membrane compared to resting platelets (159). These differences could be exploited for preferential nanoparticle uptake by engineering lipid-based nanoparticles to selectively fuse with phosphatidylserine-rich membranes (e.g., nanoparticles designed with cationic or amphiphilic lipid components). Such an approach may allow for targeted therapeutic delivery to pathogenic platelet populations while sparing healthy platelets and preserving normal hemostasis. As we advance our understanding of platelet heterogeneity and the diverse spectrum of platelet populations that are altered in disease, we can develop more precise and effective antithrombotic therapies.

Also, platelets are mediators of intercellular communication through the transfer of bioactive molecules, including mRNA, to various cell types. This capacity offers a promising avenue for delivering therapeutic mRNA to cells that are typically challenging to target using conventional delivery systems. One notable example is the transfer of platelet-derived microRNA-223 (miR-223) to vascular smooth muscle cells (VSMCs), as detailed in the study by John Hwa's group (165). In this context, miR-223 facilitated the transition of VSMCs from a synthetic/proliferating to a contractile/resting phenotype, thereby modulating tissue repair mechanisms. This finding underscores the potential of platelet-derived mRNA transfer to modulate cellular functions *in situ*, particularly in vascular injury scenarios where targeted delivery is crucial. Moreover, leveraging platelets' inherent ability to home to sites of vascular injury or inflammation could enable the precise delivery of therapeutic mRNA, minimizing off-target effects and enhancing treatment efficacy. Therefore, the generation of a variety of LNP modified PLTs is a strategy that holds significant promise for addressing conditions such as atherosclerosis, thrombosis, and other vascular pathologies where conventional delivery methods face substantial limitations.

**Figure 5:**
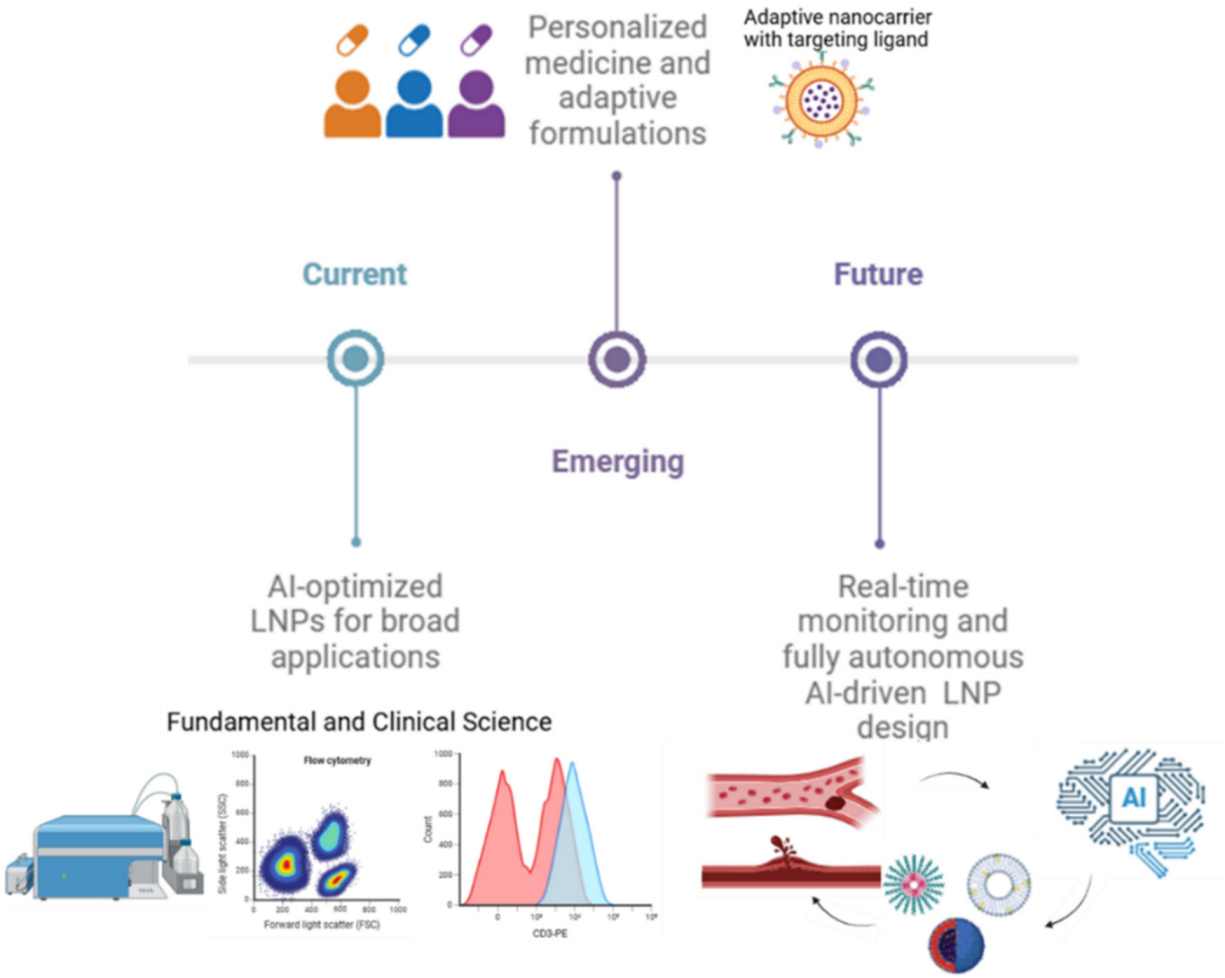
Future prospects of AI in LNP development roadmap. Current advances are supported by AI-optimized LNPs for broad applications, emerging techniques involving personalized medicine and adaptive formulations, and future goals including real-time monitoring and fully autonomous AI-driven LNP design.

AI is set to transform gene delivery by optimizing nanocarrier formulations through data-driven predictions. Algorithms will analyze parameters such as administration routes, cargo types, and *in vivo* conditions to recommend ideal LNP compositions, surface ligands, and encapsulation strategies. Machine learning models trained on experimental data can predict effective nanocarrier designs while minimizing off-target effects and immunogenicity. See Figure 5. Beyond formulation, AI can refine the delivery process by identifying optimal targeting strategies and monitoring therapeutic efficacy in real time. Reinforcement learning models will simulate physiological conditions, while generative models can propose novel nanocarrier designs. Ultimately, AI-driven automation of experimental design and high-throughput data analysis can accelerate research, reducing time and costs associated with preclinical studies. See Figure 5.

### 7.1 Industry perspectives and AI-driven manufacturing of LNPs

As LNPs continue to gain prominence in clinical applications, there has been a parallel surge in industrial interest and investment in scalable, reproducible, and quality-controlled LNP manufacturing. While academic research typically focuses on molecular design and biological efficacy, the transition to clinical and commercial success hinges on overcoming manufacturing bottlenecks. Among these are challenges in ensuring batch-to-batch consistency, scalability of microfluidic-based production, and real-time quality control of LNPs (166, 167).

Pharmaceutical companies such as Moderna and Pfizer-BioNTech have optimized LNP production through continuous-flow microfluidic systems, which enable precise control over particle size and encapsulation efficiency. However, at industrial scales, even microfluidics must be adapted to high-throughput settings. To address this, scalable platforms such as NxGen™ (Precision Nanosystems) and the NanoAssemblr® technology have been adopted for GMP-grade LNP production. These systems are capable of maintaining tight control over critical process parameters such as flow rate, lipid-to-nucleic acid ratio, and mixing time—all of which influence LNP characteristics.

A growing number of biotechnology firms and industrial research centers are leveraging AI and ML to further refine LNP formulation and production processes. AI models can be trained on large datasets comprising formulation inputs (e.g., lipid composition, molar ratios, buffer conditions) and process variables (e.g., temperature, flow rates), with outputs including particle size, polydispersity index, encapsulation efficiency, and stability. These predictive models are increasingly used to identify optimal formulations faster than traditional trial-and-error approaches. Moreover, digital twins—virtual models of manufacturing systems—are being explored to simulate and optimize production workflows in real-time. This approach enables early identification of deviations in quality and reduces manufacturing downtime. AI is also playing a role in automated fault detection, process control, and quality assurance, which are crucial for regulatory compliance and consistent therapeutic performance.

Several industry insights support these advancements. For example, the National Institute for Innovation in Manufacturing Biopharmaceuticals has published (168) strategic insights into scaling LNP production with AI-enhanced process analytical technologies. Insights from Cytiva and Precision Nanosystems also illustrate how AI-enabled formulation screening and scale-up have been critical in accelerating the development of mRNA-based vaccines and therapeutics. These efforts underscore the industrial readiness and adaptability of AI for enhancing both upstream formulation design and downstream manufacturing logistics. Integrating these industrial perspectives into academic dialogue is essential, as the ultimate goal of nanomedicine research is to achieve real-world clinical translation. Understanding the challenges and innovations in large-scale manufacturing ensures that early-stage discoveries are developed with scalability, regulatory feasibility, and patient access in mind.

## 8 Conclusion

Lipid nanoparticles have become essential for nucleic acid delivery, offering protection, targeted release, and enhanced stability. Their success in mRNA-based COVID-19 vaccines highlights their broader potential in gene editing and treating genetic and acquired diseases. In hematologic therapies, LNPs could transform treatments for hemophilia, sickle cell anemia, and thrombosis by enabling precise genetic modulation. AI-driven optimization of lipid composition and encapsulation efficiency further enhances their potential, accelerating the development of next-generation LNP systems for diverse clinical applications. Realizing LNP-based therapy requires a multidisciplinary approach, integrating materials science, pharmacology, and AI. Collaboration among academia, industry, and regulators is crucial to overcoming barriers and driving innovations that will shape the future of precision medicine.
